# Association between preserved ratio impaired spirometry and postoperative pulmonary complications in patients undergoing lung cancer resection

**DOI:** 10.3389/fmed.2026.1803143

**Published:** 2026-10-14

**Authors:** Tianran Zhou, Xingxing Sun, Jiao Zhang, Jianhua Xu, Hanqing Zhu, Mian He, Jinming Liu, Jian Guo

**Affiliations:** 1 Department of Pulmonary Function Test, Shanghai Pulmonary Hospital, School of Medicine, Tongji University, Shanghai, China; 2 Department of Cardio-Pulmonary Circulation, Shanghai Pulmonary Hospital, School of Medicine, Tongji University, Shanghai, China

**Keywords:** COPD, postoperative pulmonary complication, postoperative pulmonary complications, preserved ratio impaired spirometry, spirometry

## Abstract

**Background:**

Postoperative pulmonary complications (PPCs) are associated with adverse clinical outcomes and increased hospitalization-related costs. Preserved ratio impaired lung function (PRISm) is a lung function phenotype distinct from obstructive lung disease, and little is known about the effect of PRISm on PPCs. The aim of this study was to investigate the association between PRISm and PPCs in patients undergoing lung cancer resection.

**Methods:**

This retrospective cohort study involved patients who underwent lung cancer resection in the thoracic surgery case database between January 2019 and June 2019 at Shanghai Pulmonary Hospital. Demographics, preoperative measurements of spirometry, operative characteristics, PPCs, and length of stay were assessed.

**Results:**

The incidence of PPCs in the three groups was 36.84% in the PRISm group, 23.39% in the normal spirometry group, and 51.57% in the COPD group. Using a variable-selection strategy (forced clinical confounders plus univariable screening at *P* < 0.20), patients with PRISm were 2.07 times more likely to develop a PPC compared with normal spirometry [95% confidence interval (95% CI): 1.21–3.55; *p* = 0.008], and the relationship was not statistically different between PRISm and COPD groups [odds ratio (OR) = 0.63, 95% CI: 0.38–1.04; *p* = 0.071]. In subgroup analyses, non-restrictive PRISm was not associated with a significantly increased risk of PPCs relative to restrictive PRISm (adjusted OR = 1.32, 95% CI: 0.59–2.95; *p* = 0.494).

**Conclusions:**

Compared with normal spirometry, PRISm was independently associated with a higher risk of PPCs in patients undergoing lung cancer resection. Preoperative identification of PRISm may refine preoperative risk stratification. Candidate targeted strategies, such as preoperative pulmonary rehabilitation, lung-protective intraoperative ventilation, and intensified postoperative surveillance, warrant prospective evaluation. The risk did not differ significantly between PRISm subtypes, though this finding requires further validation.

## 1 Introduction

Lung cancer resection is associated with an increased risk of postoperative pulmonary complications (PPCs) (1). PPC has a significant impact on both patient health and health care delivery (2). It is associated with increased postoperative mortality and prolonged hospitalization (3).

Current research suggests that smoking and chronic obstructive pulmonary disease (COPD) are risk factors for PPCs (4). Preserved ratio impaired spirometry (PRISm), defined as a reduction (<80% predicted) in 1s force expiratory volume (FEV1) with a preserved FEV1 to forced vital capacity (FVC) ratio (≥0.7), is considered a precursor to COPD with a high prevalence (3%–20%) (5–7). However, this condition is sometimes overlooked because its symptoms are often not obvious (8).

Several pathophysiological mechanisms may link the PRISm phenotype to postoperative morbidity after lung resection. PRISm reflects reduced ventilatory reserve: the proportionate reduction in FEV1 and FVC leaves patients with less physiological margin to absorb the acute loss of functional parenchyma, anesthesia-related atelectasis, and chest-wall disruption caused by resection, predisposing to postoperative hypoxemia and atelectasis (5, 7). Beyond this loss of reserve, PRISm is associated with distal airway and parenchymal pathology—terminal bronchiolar loss, small-airway remodeling, and early emphysematous change—that may compromise alveolar recruitment and collateral ventilation in the remaining lung (20–22). PRISm has also been linked to reduced resting oxygen saturation; chronic hypoxia can activate pro-inflammatory signaling and microvascular endothelial dysfunction, amplifying susceptibility to postoperative infection and acute lung injury (23, 24). In addition, PRISm clusters with metabolic and cardiovascular comorbidities and chronic respiratory symptoms; together with impaired cough effectiveness after thoracotomy, these factors promote sputum retention and respiratory infection after thoracic surgery (5, 7). These mechanisms provide a biological rationale for an elevated PPC risk in PRISm beyond that conferred by normal spirometry.

The association between PRISm and the occurrence of PPCs in patients undergoing surgery for lung cancer has not yet been determined. Therefore, it is crucial to determine whether PRISm is a potential risk factor for PPCs. We hypothesized that PRISm is independently associated with an increased risk of PPCs in patients undergoing lung cancer resection. Specifically, we posited that the risk of PPCs in patients with PRISm would be significantly higher than in those with normal spirometry, and potentially comparable to the risk observed in patients with COPD.

The aim of this study was to investigate the impact of PRISm on the incidence of PPCs among patients undergoing lung cancer surgery. If this association is confirmed, preoperative identification of the PRISm phenotype could have clinical implications for perioperative management. For instance, patients with PRISm might benefit from a stage-specific approach comprising: (i) preoperative structured pulmonary rehabilitation and inspiratory muscle training, intensified smoking-cessation support, optimization of coexisting airway disease (e.g., inhaled bronchodilator therapy where clinically indicated), and incentive-spirometry education; (ii) intraoperative preference for minimally invasive approaches where oncologically appropriate, careful consideration of resection extent, and lung-protective one-lung ventilation; and (iii) postoperative intensified surveillance with a lower threshold for chest imaging and arterial blood gas analysis, early mobilization with aggressive chest physiotherapy, and timely escalation of respiratory support. We emphasize, however, that this proposed approach is a hypothesis generated by our findings rather than an intervention directly supported by the present observational study, and its effectiveness should be tested in prospective studies.

## 2 Methods

### 2.1 Study design and population

This retrospective observational cohort study was conducted in the thoracic surgery department of a large hospital. The database of all thoracic surgery cases at Shanghai pulmonary Hospital of Tongji University from January 2019 to June 2019 was queried for patients who underwent lung cancer resection. Inclusion criteria were: age ≥18 years and availability of reliable preoperative pulmonary function test (PFT) results. Exclusion criteria were: a combination of asthma, interstitial lung disease, or other lung diseases; a history of thoracic surgery. Patients with incomplete data were also excluded.

These pulmonary comorbidities were excluded deliberately. Asthma, interstitial lung disease, and other chronic pulmonary conditions independently alter both spirometric patterns and PPC risk; retaining them would confound the PRISm-PPC association and contaminate phenotype classification (for example, interstitial lung disease can produce a spirometric pattern overlapping with PRISm while reflecting true parenchymal restriction, and asthma can cause airflow limitation overlapping with COPD definitions). Excluding these conditions therefore isolates the PRISm phenotype as cleanly as spirometry alone allows and protects internal validity. The consequences of this selection for external validity are addressed in the Discussion.

### 2.2 Pulmonary function testing and quality assurance

PFTs (Jaeger Toennies spirometer, Germany) were performed by eight certified and experienced pulmonary function technicians in accordance with American Thoracic Society (ATS) guidelines (9). All test results were reviewed and confirmed by a pulmonologist before being entered into the final report. Reliable test results were used for patient grouping. Reliable PFT results were defined: at least three acceptable spirograms with reproducible FEV_1_ and FVC values (≤0.15 L difference between the two highest readings) were required.

Laboratory quality assurance included daily calibration verification with a 3-L syringe and standardized bronchodilator testing procedures. Because all PFTs were performed during routine preoperative clinical care in 2019—before this study was conceived—the technicians were unaware of the study hypothesis and of any subsequent group assignment, effectively blinding them to the study question.

The participants were divided into 3 groups based on preoperative PFT results. PRISm (*n* = 171) was defined as FEV1/FVC more than or equal to 0.7 and FEV1% < 80% predicted after bronchodilator inhalation, and normal spirometry (*n* = 171) was defined as FEV1/FVC more than or equal to 0.7 and FEV1% ≥ 80% predicted after bronchodilator inhalation [This group was 1:1 matched to the PRISm group by age, sex, and body mass index (BMI) to reduce confounding by baseline demographic factors]. COPD (*n* = 223) was defined as FEV1/FVC less than 0.7 after bronchodilator inhalation (10).

PRISm was categorized into restrictive PRISm (FEV1/FVC more than or equal to 0.7, FEV1 < 80% predicted and FVC < 80% predicted after bronchodilator inhalation) and non-restrictive PRISm (FEV1/FVC ≥ 0.7, FEV1 < 80% predicted and FVC ≥ 80% predicted after bronchodilator inhalation) (11) in the subgroup analysis. The definition of small airway dysfunction (SAD) was based on previous studies, using spirometric indices such as maximum expiratory flow rates evaluated at the usual intervals of 25% of exhaled forced vital capacity (MEF25), maximum expiratory flow rates evaluated at the usual intervals of 50% of exhaled forced vital capacity (MEF50) and maximum mid-expiratory flow (MMEF), which are acknowledged as the most feasible and practical markers (12, 13).

### 2.3 Outcomes and data collection

According to the European Perioperative Clinical Outcome (EPCO) definitions (14), PPCs include respiratory infection, aspiration pneumonitis, pleural effusion, pneumothorax, respiratory failure, atelectasis, and bronchospasm.

For this study, each component was adjudicated using the following explicit operational criteria: (i) respiratory infection—treatment with antibiotics for a suspected respiratory infection plus at least one of the following: new or changed sputum, new or changed lung opacities, fever, or white blood cell count >12 × 10^9^/L; (ii) aspiration pneumonitis—acute lung injury following the inhalation of regurgitated gastric contents; (iii) pleural effusion—chest radiograph showing blunting of the costophrenic angle, loss of the sharp silhouette of the ipsilateral hemidiaphragm in the upright position, displacement of adjacent anatomical structures, or, in the supine position, a hazy opacity in one hemithorax with preserved vascular markings; (iv) pneumothorax—air in the pleural space with no vascular bed surrounding the visceral pleura on chest imaging; (v) respiratory failure—postoperative PaO2 < 60 mmHg on room air, PaO2/FiO2 ratio < 300 mmHg, or SpO2 < 90% requiring oxygen therapy; (vi) atelectasis—lung opacification with shift of the mediastinum, hilum, or hemidiaphragm toward the affected area and compensatory overinflation of the adjacent non-atelectatic lung; and (vii) bronchospasm—newly detected expiratory wheezing treated with bronchodilators. PPCs were abstracted from the electronic medical record by investigators who were blinded to patients’ PFT group assignments; because group assignment was performed after data collection, outcome assessment effectively preceded exposure classification. The presence of any PPC was determined by a retrospective review of electronic medical records, including daily progress notes, imaging reports, and discharge summaries. The assessment period for PPCs covered the entire postoperative hospital stay, from the day of surgery until the date of discharge.

8th edition staging guidelines by the American Joint Committee on Cancer (AJCC) (2017) were used for the purpose of staging. Data collected included demographics, preoperative smoking status, comorbidities, lung function, American Society of Anesthesiologists (ASA) score, tumor characteristics, and intraoperative/postoperative data. Postoperative hospital stay, defined as the time of hospitalization after the date of surgery, was pre-specified as a secondary outcome. As a postoperative consequence plausibly influenced by both lung function and PPCs, it was not eligible as a confounder and was never entered into any multivariable model. All patients underwent general anesthesia with double-lumen tube intubation and one-lung ventilation under a standardized institutional protocol.

Ethical approval for this study was obtained from the Ethics Commission of Shanghai pulmonary Hospital of Tongji University (L20-236-1).

### 2.4 Statistical analysis

Results are expressed as mean ± standard deviation (SD) or median (interquartile range) for continuous variables and numbers (percentages) for categorical variables. Comparisons among the three groups (PRISm, normal spirometry, and COPD) were performed using the Kruskal–Wallis test for continuous variables and the chi-squared test or Fisher's exact test for categorical variables. For the 1:1 matched PRISm and normal spirometry groups, covariate balance was assessed using standardized mean differences (SMD). An absolute SMD < 0.2 was considered indicative of adequate balance.

Multivariable logistic regression was used to assess the independent association between pulmonary function phenotypes and PPCs. Variables were selected using a combined clinical and statistical strategy: core, pre-specified confounders (age, sex, BMI, history of smoking, and pack-years), chosen *a priori* on clinical grounds and guided by a directed acyclic graph (DAG) of the assumed causal relationships among lung-function phenotype, patient/tumor/surgical factors, and PPCs, were forced into the models; in addition, covariates with *P* < 0.20 in univariable analyses were retained, a more liberal threshold intended to avoid omitting meaningful confounders. Under this strategy, the final PRISm-vs.-normal model additionally retained hypertension, type of surgery, operative duration, largest tumor dimension, tumor location, pathology, and NSCLC staging; the PRISm-vs.-COPD model additionally retained surgical approach, type of surgery, operative duration, blood loss, largest tumor dimension, tumor location, pathology, and NSCLC staging. Variables with zero-cell distributions across groups were excluded to ensure model convergence. Multicollinearity among independent variables was assessed using the Variance Inflation Factor (VIF) prior to modeling, with a VIF threshold of 5 (all VIFs were below 5 in the final models). Complete univariable analysis results and event distribution data are provided in Supplementary Table S1.

Missing data were handled by complete-case analysis: patients with missing grouping variables, key covariates, or outcome data were excluded before matching and modeling, and the number excluded for incomplete data is reported in the study flow diagram (Figure 1). Analyses were conducted in SPSS 26.0. The statistical significance threshold was set as two-sided *p* < 0.05.

**Figure 1:**
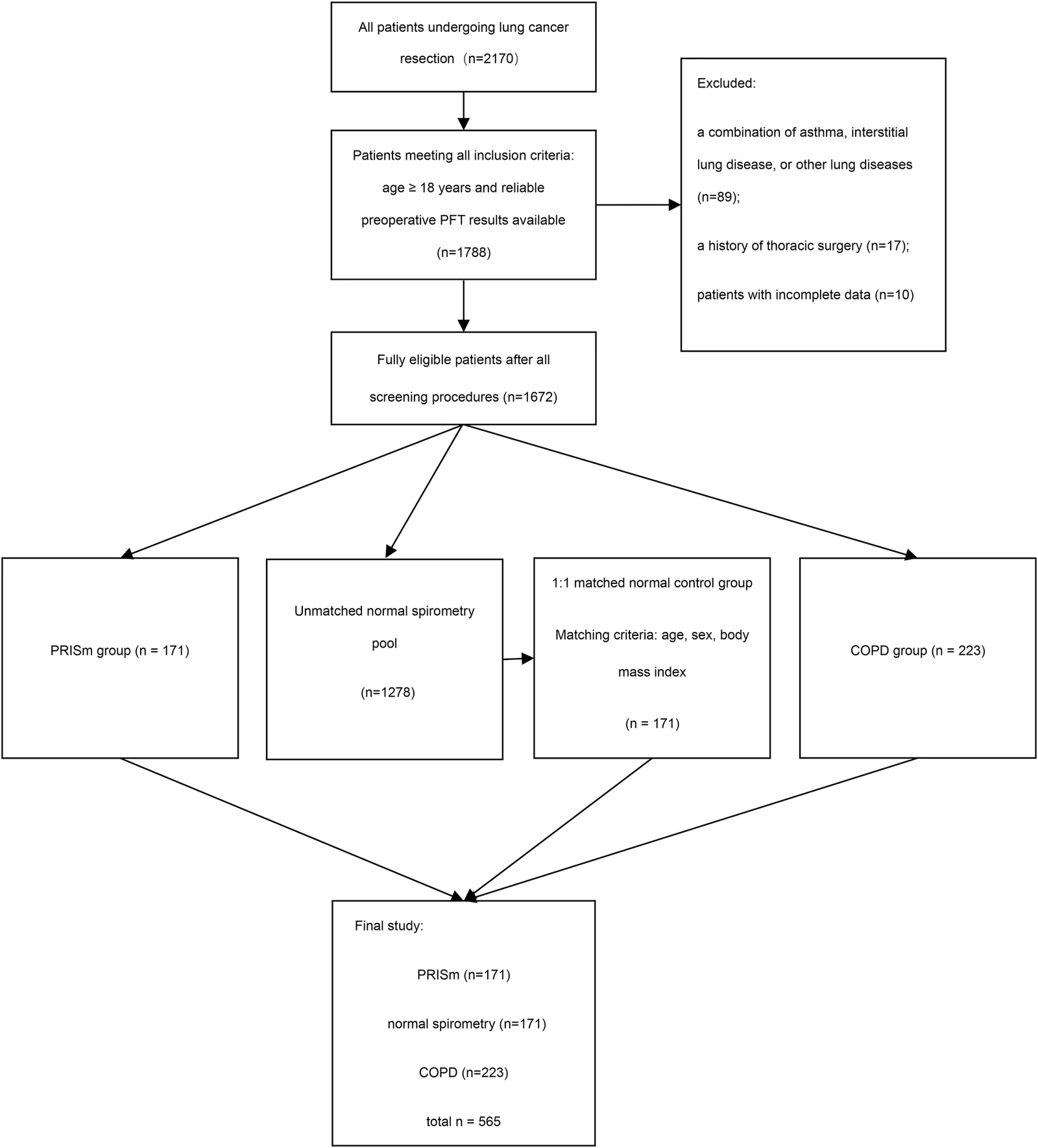
Flow diagram of patient selection and group allocation.

## 3 Results

### 3.1 Baseline characteristics and lung function

A total of 565 patients were included in the final analysis, comprising 171 in the PRISm group, 171 in the matched normal spirometry group, and 223 in the COPD group (Figure 1). The baseline demographic, clinical, and operative characteristics are summarized in Table 1.

**Table 1:** Baseline characteristics of PRISm, normal spirometry and COPD groups.

Characteristics	PRISm (*n* = 171)	Normal spirometry (*n* = 171)	COPD (*n* = 223)	Overall *P*	SMD
Age (years), median [IQR]	63.00 [13.00]	63.00 [13.00]	65.00 [10.00]	**0.001**	<0.001
BMI (kg/m^2^), median [IQR]	24.73 [4.3]	24.56 [4.18]	24.11 [3.69]	0.139	0.054
Male, *n* (%)	63 (36.84%)	53 (31.00%)	159 (71.30%)	**<0.001**	0.124
Pack-year, mean ± SD	3.95 (13.52)	2.33 (8.59)	10.89 (22.27)	**<0.001**	0.143
History of smoking, *n* (%)	17 (9.94%)	15 (8.77%)	57 (25.56%)	**<0.001**	0.040
Hypertension, *n* (%)	51 (29.82%)	36 (21.05%)	56 (25.11%)	0.174	0.203
Diabetes, *n* (%)	23 (13.45%)	10 (5.85%)	10 (4.48%)	**0.002**	0.259
Surgical approach, *n* (%)				**0.018**	0.058
VATS/RATS	170 (99.42%)	171 (100.00%)	215 (96.41%)		
Open thoracotomy	1 (0.58%)	0 (0.00%)	8 (3.59%)		
Type of surgery, *n* (%)				0.127	—
Lobectomy	101 (59.06%)	97 (56.73%)	129 (57.85%)		
Sub-lobar resection	63 (36.84%)	74 (43.27%)	83 (37.22%)		
Sleeve lobectomy	4 (2.34%)	0 (0.00%)	8 (3.59%)		
Pneumonectomy	3 (1.75%)	0 (0.00%)	3 (1.35%)		
Operative duration (h), mean ± SD	1.88 (0.64)	1.72 (0.68)	1.97 (0.72)	**0.001**	0.242
Blood loss (mL), mean ± SD	62.02 (86.09)	53.19 (64.38)	73.79 (116.84)	**0.002**	0.116
Postoperative hospital stay (days), mean ± SD	3.89 (1.95)	3.50 (1.65)	4.44 (2.43)	**<0.001**	0.216
Largest tumor dimension (mm), median [IQR]	15.60 [14.00]	13.30 [11.50]	17.00 [15.00]	**0.004**	0.242
Tumor location, *n* (%)				0.050	—
RUL	70 (40.94%)	65 (38.01%)	67 (30.04%)		
RML	16 (9.36%)	20 (11.70%)	13 (5.83%)		
RLL	32 (18.71%)	29 (16.96%)	38 (17.04%)		
LUL	32 (18.71%)	36 (21.05%)	64 (28.70%)		
LLL	21 (12.28%)	21 (12.28%)	41 (18.39%)		
Pathology, *n* (%)				**<0.001**	—
Adenocarcinoma	147 (85.96%)	157 (91.81%)	155 (69.51%)		
Squamous Cell	16 (9.36%)	9 (5.26%)	48 (21.52%)		
Small Cell	0 (0.00%)	1 (0.58%)	20 (8.97%)		
others	8 (4.68%)	4 (2.34%)	0 (0.00%)		
NSCLC staging, *n* (%)				**0.026**	—
Tis/I	147 (85.96%)	155 (90.64%)	197 (88.34%)		
II	23 (13.45%)	10 (5.85%)	17 (7.62%)		
III/IV	1 (0.58%)	6 (3.51%)	9 (4.04%)		
ASA score ≦ 2, *n* (%)	171 (100.00%)	171 (100.00%)	222 (99.55%)	0.465	0.000
PPCs, *n* (%)	63(36.84%)	40(23.39%)	115(51.57%)	**<0.001**	—

PRISm, preserved ratio impaired spirometry; COPD, chronic obstructive pulmonary disease; BMI, body mass index; 1 pack-year is defined as 20 cigarettes smoked every day for 1 year. VATS, video-assisted thoracic surgery; RATS, robotic-assisted thoracic surgery; RUL, right upper lobe; RML, right middle lobe; RLL, right lower lobe; LUL, left upper lobe; LLL, left lower lobe; NSCLC, non-small cell lung cancer; ASA, *American Society of Anesthesiologists*; PPC, postoperative pulmonary complication; SMD, standardized mean difference. PPCs and postoperative hospital stay were presented for descriptive purposes only and were not included in multivariable models.

Overall *P* value from Kruskal–Wallis *H* test, or chi-square test for comparison across all three groups.

For continuous variables, SMD was calculated as the absolute difference in means divided by the pooled standard deviation. For binary variables, SMD was calculated as the absolute difference in proportions divided by the pooled standard deviation. For polytomous variables, a single SMD is not reported; instead, the distribution is presented descriptively. SMD < 0.2 was considered indicative of adequate balance between the matched PRISm and normal spirometry groups.

Bold values indicate statistically significant results (*p* < 0.05).

After 1:1 matching, the PRISm and normal spirometry groups were well balanced on the three matching variables (age, sex, BMI), with all standardized mean differences (SMD) < 0.2 (age <0.001, sex 0.124, BMI 0.054). Despite this overall balance, several other baseline characteristics showed residual imbalance (SMD >0.2), including diabetes (13.45% vs. 5.85%; SMD: 0.259), operative duration (1.88 ± 0.64 vs. 1.72 ± 0.68 h; SMD 0.242), largest tumor dimension (median 15.60 vs. 13.30 mm; SMD: 0.242). Hypertension showed borderline imbalance (SMD: 0.203). The distribution of NSCLC staging also differed between the groups. As expected, lung function indices differed substantially between the PRISm and normal spirometry groups: FEV_1_, FVC, and all measures of small airway function were notably lower in the PRISm group (Table 2).

**Table 2:** Lung function of PRISm, normal spirometry and COPD groups.

Lung function	PRISm (*n* = 171)	normal spirometry (*n* = 171)	COPD (*n* = 223)	Overall *P*
FEV1, L	1.73 [0.63]	2.29 [0.63]	1.80 [0.64]	**<0.001**
FEV1% of predicted, %	74.30 [6.93]	98.10 [15.80]	74.50 [20.17]	**<0.001**
FEV1/FVC, %	77.64 [9.26]	82.21 [8.09]	64.94 [7.70]	**<0.001**
FVC, L	2.24 [0.81]	2.79 [0.72]	2.88 [0.90]	**<0.001**
FVC% of predicted, %	75.00 [10.50]	96.20 [17.20]	92.30 [19.33]	**<0.001**
MEF25, L/s	0.59 [0.38]	0.91 [0.55]	0.39 [0.21]	**<0.001**
MEF25% of predicted, %	51.00 [26.20]	85.60 [38.70]	36.50 [16.90]	**<0.001**
MEF50, L/s	1.98 [1.13]	3.19 [1.56]	1.26 [0.58]	**<0.001**
MEF50% of predicted, %	59.00 [23.60]	99.70 [41.90]	39.30 [17.67]	**<0.001**
MMEF, L/s	1.47 [0.73]	2.39 [1.19]	0.94 [0.50]	**<0.001**
MMEF% of predicted, %	49.40 [20.60]	81.20 [32.70]	32.65 [13.20]	**<0.001**

Data are presented as median [interquartile range].

PRISm, preserved ratio impaired spirometry; COPD, chronic obstructive pulmonary disease; FEV1 = forced expiratory volume in one second; FVC = forced vital capacity; MEF25, maximum expiratory flow rates evaluated at the usual intervals of 25% of exhaled forced vital capacity; MEF50, maximum expiratory flow rates evaluated at the usual intervals of 50% of exhaled forced vital capacity; MMEF, maximum mid-expiratory flow.

Overall *P* value from Kruskal–Wallis *H* test for comparison across all three groups.

Bold values indicate statistically significant results (*p* < 0.05).

Compared with the COPD group, patients in the PRISm group were younger, had a substantially lower proportion of males (36.84% vs. 71.30%), and a markedly less pronounced smoking history (9.94% vs. 25.56% with any smoking; mean pack-years 3.95 vs. 10.89). Conversely, diabetes was more prevalent in the PRISm group (13.45% vs. 4.48%). Operative duration, blood loss, and largest tumor dimension were numerically higher in the COPD group, with PRISm patients generally exhibiting intermediate values between the normal spirometry and COPD groups. Open thoracotomy was rare in the PRISm group (0.58%) but somewhat more common in the COPD group (3.59%). The distribution of pathology types also differed, with PRISm patients showing a higher proportion of adenocarcinoma (85.96% vs. 69.51%) and correspondingly lower proportions of squamous cell carcinoma and other histological types. NSCLC staging distribution similarly varied between the two groups. In contrast, BMI, hypertension, type of surgery, and ASA score were broadly comparable across groups. Regarding lung function, FEV_1_/FVC and all small airway function indices were notably higher in the PRISm group than in the COPD group, whereas FVC and FVC% predicted were notably lower. FEV_1_ and FEV_1_% predicted were comparable between the two groups (Table 2).

### 3.2 Comparison of PPCs in PRISm, normal spirometry, and COPD groups

The overall incidence of PPCs differed significantly across the three groups. The rates were 36.84% in the PRISm group, 23.39% in the normal spirometry group, and 51.57% in the COPD group (Table 1). Unadjusted comparisons showed that the PPCs incidence was significantly higher in the PRISm group than in the normal spirometry group (*p* = 0.007) and significantly lower than in the COPD group (*p* = 0.004). Under the variable-selection strategy (forced inclusion of age, sex, BMI, smoking history, and pack-years, plus covariates with *P* < 0.20 in univariable analyses), multivariable logistic regression analysis confirmed that patients with PRISm had a significantly higher risk of developing a PPC compared to those with normal spirometry [adjusted odds ratio (OR) = 2.07, 95% confidence interval (CI): 1.21–3.55; *p* = 0.008] (Table 3). When compared to the COPD group, the risk of PPCs in the PRISm group was not statistically different after adjustment (OR = 0.63, 95% CI: 0.38–1.04; *p* = 0.071) (Table 4). Complete univariable regression results and PPC event distributions for all included variables are summarized in Supplementary Table S1.

**Table 3:** Comparison of PPCs in PRISm and normal spirometry group.

Groups	PPC	
	OR (95% CI)	*P* value
Normal spirometry (*n* = 171)	Reference	
PRISm (*n* = 171)	2.07 (1.21, 3.55)	**0.008**

PPC, postoperative pulmonary complication; PRISm, preserved ratio impaired spirometry; OR, odds ratio; CI, confidence interval.

Adjusted for age, sex, BMI, history of smoking, pack-years (forced), plus hypertension, type of surgery, operative duration, largest tumor dimension, tumor location, pathology, and NSCLC staging (univariable *P* < 0.20); all VIF < 5.

Bold values indicate statistically significant results (*p* < 0.05).

**Table 4:** Comparison of PPCs in PRISm and COPD group.

Groups	PPC	
	OR (95% CI)	*P* value
COPD (*n* = 223)	Reference	
PRISm (*n* = 171)	0.63 (0.38, 1.04)	0.071

PPC, postoperative pulmonary complication; PRISm, preserved ratio impaired spirometry; OR, odds ratio; CI, confidence interval.

Adjusted for age, sex, BMI, history of smoking, pack-years (forced), plus surgical approach, type of surgery, operative duration, blood loss, largest tumor dimension, tumor location, pathology, and NSCLC staging (univariable *P* < 0.20); all VIF < 5.

Postoperative hospital stay, analyzed as a secondary outcome, differed significantly across the three groups (PRISm 3.89 ± 1.95 days vs. normal spirometry 3.50 ± 1.65 days vs. COPD 4.44 ± 2.43 days; Kruskal–Wallis *P* < 0.001).

### 3.3 Subgroup analysis

Exploratory subgroup analysis was conducted within the PRISm group. After full adjustment for confounders, patients with non-restrictive PRISm showed no clear excess in PPC risk compared with those with restrictive PRISm, but the difference did not reach statistical significance (adjusted OR = 1.32, 95% CI: 0.59–2.95; *P* = 0.494) (Table 5). For the SAD subgroup analysis, no statistically significant difference in PPC incidence was observed between patients with and without SAD in either unadjusted or adjusted models, with no apparent trend of elevated risk (all *P* > 0.05) (Table 6). All internal subgroup analyses were exploratory and hypothesis-generating, limited by the small sample size of PRISm subtype groups.

**Table 5:** Correlation of non-restrictive PRISm with PPC compared to restrictive PRISm.

Groups	OR (95% CI) *P* value	
	Model 0	Model 1
Restrictive PRISm (*n* = 127)	Reference	Reference
Non-restrictive PRISm (*n* = 44)	1.26 (0.62, 2.55) 0.517	1.32 (0.59, 2.95) 0.494

PRISm, preserved ratio impaired spirometry; PPC, postoperative pulmonary complication; OR, odds ratio; CI, confidence interval.

Model 0: adjusted for none.

Model 1: adjusted for age, sex, BMI, history of smoking, pack-years (forced), plus hypertension, type of surgery, largest tumor dimension, tumor location, and NSCLC staging (univariable *P* < 0.20).

**Table 6:** Correlation of PRISm-SAD with PPC compared to PRISm-nonSAD.

Groups	OR (95% CI) *P* value	
	Model 0	Model 1
PRISm-nonSAD (*n* = 40)	Reference	Reference
PRISm-SAD (*n* = 131)	1.11 (0.53, 2.33) 0.783	1.16 (0.49, 2.74) 0.729

PRISm, preserved ratio impaired spirometry; SAD, small airway dysfunction; PPC, postoperative pulmonary complication; OR, odds ratio; CI, confidence interval.

Model 0: adjusted for none.

Model 1: adjusted for age, sex, BMI, history of smoking, pack-years (forced), plus hypertension, type of surgery, largest tumor dimension, tumor location, and NSCLC staging (univariable *P* < 0.20).

## 4 Discussion

Our study confirmed that the incidence of developing a PPC following thoracic surgery was common. To the best of our knowledge, there is no study on the association between PRISm and PPCs in lung cancer resection. In this study, the incidence of PPCs in the PRISm group was 36.84%, and the risk of developing a PPC was significantly higher in patients with PRISm than in the normal spirometry group.

Thoracic surgery is associated with a higher risk of PPCs and the incidence (19%–59%) is much higher than in upper abdominal surgery (16%–17%) or lower abdominal surgery (0%–5%) (15). PPC reduces overall survival at 6 months postoperatively and is one of the leading causes of postoperative mortality (accounting for up to 84% of all deaths) (1, 16). Identifying risk factors for PPCs is critical in providing evidence for early assessment and optimization of perioperative interventions that minimize the risk of PPCs and improve patient prognosis (17). Our research has revealed that patients with PRISm had significantly higher incidence of PPCs, longer operative duration, and postoperative hospital stay. These can increase the health burden on patients and the medical burden on society. Previous studies have found multiple factors to be associated with an increased incidence of PPCs, such as age, smoking, COPD, BMI, type of surgery, duration of surgery, surgical site, and intraoperative bleeding volume (17, 18). After adjustment for potential confounding factors, the risk of PPCs remained significantly higher in the PRISm group than in the normal group in this study. This suggested that PRISm was independently associated with an increased risk of PPCs in patients undergoing lung cancer resection, and that surgeons should be aware of patients with this phenotype.

PRISm is a weakly described respiratory condition with unclear cause and prognosis. Recent investigation has indicated that PRISm may be related with the progress of COPD (6). It's probably an early physiological state of COPD (19). Previous studies have found COPD to be an independent risk factor for developing PPCs (2). The results of the analysis in this study showed there was no statistically significant difference in the incidence of PPCs between the PRISm and COPD groups after adjusting for potential confounders. Although PRISm and COPD are physiologically distinct, with COPD defined by airflow obstruction and PRISm by a proportionate reduction in FEV1 and FVC with a preserved ratio, their similar postoperative risk can be understood through convergence on a final common pathway of respiratory decompensation. Both phenotypes reduce preoperative ventilatory reserve, albeit by different routes: in COPD, expiratory flow limitation, dynamic hyperinflation, and gas trapping reduce effective ventilation, whereas in PRISm, small lung volumes and distal airway-alveolar pathology shrink the ventilatory reserve available after parenchymal resection. Because lung resection removes a fixed quantum of function, PPC risk depends on the reserve remaining relative to the surgical insult, which is diminished in both phenotypes. Both phenotypes also involve distal airway pathology: small-airway loss, chronic inflammation, and impaired mucociliary clearance are well established in COPD (20–22), whereas in PRISm, increased airway wall thickness has been documented (23) and small-airway dysfunction is highly prevalent in the population (12); these changes promote sputum retention, atelectasis, and postoperative infection. In addition, PRISm and COPD are both associated with reduced resting oxygen saturation and with cardiovascular and metabolic comorbidity burdens that independently raise PPC risk and blunt physiological compensation (5, 7, 23). Notably, the adjusted point estimate for PRISm vs. COPD (OR: 0.63, 95% CI: 0.38–1.04) trends toward a lower risk in PRISm, which is compatible with the additional burden of established airflow obstruction in COPD, although the wide confidence interval includes equivalence and reflects the limited power of this contrast.

The advancement of COPD seems to result from a combination of airway constriction and substantial distal airway loss, leading to increased resistance in the small airways (20, 21). It can be speculated that the elevated PPCs observed in PRISm patients might originate from compromised alveolar expansion associated with progressive terminal bronchiolar degeneration during initial COPD pathogenesis (22). Moreover, chronic inflammatory responses and structural reorganization of distal small airways alongside neighboring alveolar tissues could amplify susceptibility to PPCs (21). COPDGene cohort analyses have identified a correlation between PRISm and reduced resting oxygen saturation (23). This chronic hypoxic state potentially initiates a pathophysiological cascade involving pro-inflammatory cytokine activation and microvascular endothelial damage, ultimately elevating PPCs risk in PRISm patients through recruiting immune cells (24). In addition, it is worth noting that the occurrence of PPCs after thoracic surgery is associated with a poorer long-term prognosis (2). Therefore, there is a need for better management of patients with PRISm during lung resection to improve prognosis.

Clinically, our findings raise the possibility that preoperative identification of PRISm could inform a stage-specific management approach rather than generic vigilance. We emphasize that such a bundle represents a potential clinical implication and a hypothesis generated by our data, rather than an intervention directly supported by this observational study; its components might include: preoperatively, structured pulmonary rehabilitation and inspiratory muscle training, intensified smoking-cessation support, optimization of coexisting airway disease (e.g., inhaled bronchodilator therapy where clinically indicated), and incentive-spirometry education; intraoperatively, preference for minimally invasive resection where oncologically appropriate, careful titration of resection extent, and lung-protective one-lung ventilation with low tidal volume, individualized PEEP, and recruitment maneuvers; postoperatively, intensified surveillance with a lower threshold for chest imaging and arterial blood gas analysis, early mobilization with aggressive chest physiotherapy, and timely escalation to high-flow nasal oxygen or noninvasive ventilation. Prospective trials are needed to test whether such PRISm-targeted bundles reduce PPC incidence before they can be recommended for routine practice.

Previous studies have indicated that PRISm can be categorized according to the presence of restrictive abnormalities (25). Non-restrictive PRISm is independently associated with the risk of developing airflow obstruction and is more likely to be underestimated in clinical practice than restrictive PRISm (11). It does not meet the traditional criteria for obstructive and restrictive ventilation abnormalities and may be a precursor to COPD, warranting increased surveillance (11). Non-restrictive PRISm is associated with a history of asthma and smoking, and its pathophysiology may include intrapulmonary factors such as airway inflammation, emphysema, and bronchial hyperresponsiveness (11).

In the present exploratory subgroup analysis of PRISm patients, we found no statistically significant difference in the risk of PPCs between non-restrictive and restrictive PRISm. The crude OR was 1.26 (95% CI: 0.62–2.55, *P* = 0.517) before adjustment, and 1.32 (95% CI: 0.59–2.95, *P* = 0.494) after adjusting for confounders. The adjusted estimate shows no clear excess risk in the non-restrictive subtype, although the wide confidence interval does not exclude a clinically meaningful difference. The negative result is most likely attributable to the limited sample size within the PRISm group, with only 44 patients in the non-restrictive PRISm subgroup, leading to insufficient statistical power and a potential type II error. Currently, our findings do not support the conclusion that non-restrictive PRISm represents a high-risk PPC subtype within the PRISm population. However, given the observed trend toward increased risk of non-restrictive PRISm reported in prior literature, appropriate perioperative attention to this subtype remains clinically reasonable. The risk of model overfitting was evaluated via the events per variable (EPV) ratio. The EPV was approximately 5.4 for the PRISm vs. normal spirometry model and 8.5 for the PRISm vs. COPD model. While the EPV of the control comparison is below the traditional threshold of 10, it meets the more recently proposed acceptable threshold of 5 for knowledge-driven variable selection (27). Our pre-specified variable selection strategy and merging of low-frequency subgroups further mitigated overfitting risk. The directional conclusion of the core association between PRISm and PPC risk remains robust, though effect estimates for low-frequency covariate subgroups should be interpreted with caution. Additionally, subgroup analyses within the PRISm group are exploratory in nature and should be interpreted as hypothesis-generating findings.

### 4.1 Limitations

Several limitations of this study should be acknowledged. First, its retrospective, single-center design inherently limits the generalizability of our findings. Relatedly, we excluded patients with asthma, interstitial lung disease, and other pulmonary comorbidities to isolate the PRISm phenotype and protect internal validity; however, in real-world thoracic practice such comorbidities are common and may themselves modify PPC risk, so our effect estimates strictly apply to lung-cancer surgical candidates without these conditions, and the magnitude and ranking of PRISm-associated risk in unselected populations may differ. Inclusive prospective cohorts are a necessary next step. Second, the definition of PRISm was based solely on spirometry, without measurement of total lung capacity (TLC). Our sub-classification of “restrictive PRISm” (based on FVC <80% predicted) is spirometric and speculative. The findings related to this subgroup must be interpreted with extreme caution. Future prospective studies incorporating full pulmonary function tests, including plethysmography for TLC measurement, are essential to clarify the underlying pathophysiology of PRISm subtypes and their impact on postoperative outcomes. Third, our assessment of SAD relied on spirometric indices. Therefore, the findings regarding SAD in our cohort should be considered exploratory. Although various methods exist for assessing small airway function, spirometry has emerged as the most widely used approach due to its feasibility and practicality. Our decision to employ spirometry was driven by these distinct advantages in terms of efficiency and operability within a large cohort (12). Future studies incorporating forced oscillation technique or other advanced modalities are warranted to further validate our findings. Fourth, our analysis was based on % predicted values for spirometric parameters rather than the currently recommended Z-scores, as per the 2022 ERS/ATS technical standards (26). This was due to the historical nature of the data from 2019. The use of % predicted values, particularly a fixed threshold of 80%, may lead to misclassification of lung function, especially in younger and older individuals. This limitation underscores the need for future studies to utilize Z-scores for more accurate classification. Notably, the 2025 GOLD Report (https://goldcopd.org/2025-gold-report/) defines PRISm based on a fixed FEV_1_/FVC cutoff (≥0.70) and reduced FEV_1_ (<80% predicted). Fifth, the inclusion period from January to June 2019 may introduce seasonal bias, as respiratory complications could be more prevalent during winter months. Future studies should encompass multi-year data to account for seasonal variations. Sixth, the incidence of PPCs is highly dependent on the definition used. Our study utilized the EPCO definitions. However, future studies should incorporate sensitivity analyses across multiple definitions to strengthen the robustness of the findings. Besides, our primary endpoint was restricted to in-hospital postoperative pulmonary complications. Future prospective studies incorporating 30-day post-discharge follow-up will help further delineate the full profile of postoperative pulmonary morbidity across different lung function phenotypes. Seventh, structured intraoperative ventilation parameters and cumulative fluid balance were not retrievable from the anesthesia record system used during the study period; although anesthetic technique was uniform across groups, unmeasured variation in ventilation and fluid management may have caused residual confounding. Eighth, our variable-selection strategy combined forced clinical confounders with univariable screening at *P* < 0.20; even at this liberal threshold, *P*-value-based screening may omit weak but true confounders, and DAG-informed selection depends on the assumed causal structure. Finally, the study lacked longitudinal follow-up, which precluded evaluation of long-term prognosis in patients with PRISm after lung cancer resection.

## 5 Conclusion

The findings of our study demonstrate that PRISm is associated with an increased risk of postoperative pulmonary complications in patients undergoing lung cancer resection. Exploratory subgroup analysis within the PRISm cohort showed no statistically significant difference in PPC risk between restrictive and non-restrictive PRISm subtypes. These results suggest that routine screening for the PRISm phenotype before lung resection may improve preoperative risk stratification. A stage-specific perioperative management bundle for patients with PRISm is a potential clinical implication of our findings; whether it can prevent or mitigate pulmonary complications should be examined in large-scale prospective studies.

## Data Availability

The datasets used and/or analyzed during the current study are available from the corresponding author on reasonable request.
