# Supplementary material for: Association between preserved ratio impaired spirometry and postoperative pulmonary complications in patients undergoing lung cancer resection
Source: Front Med (Lausanne). 2026 Oct 14;13:1803143. doi: 10.3389/fmed.2026.1803143 (PMC13713789; doi:10.3389/fmed.2026.1803143)
Supplement: Supplementary file 1 [file Table1.docx]

**Supplementary materials**

Table 1 Correlation of non-restricted PRISm with PPC compared to COPD

| Groups | PPC | |
| --- | --- | --- |
|  | OR (95%CI) | *P* value |
| COPD | Reference |  |
| Non-restricted PRISm | 1.104 (0.501,2.433) | 0.806 |

Abbreviations: PRISm, preserved ratio impaired spirometry; PPC, postoperative pulmonary complication; COPD, chronic obstructive pulmonary disease; OR, odd ratio; CI, confidence interval.

Adjusted for sex, age, BMI, history of smoking, pack-years, hypertension, diabetes, largest tumor dimension, tumor location, surgical approach, type of surgery, operative duration, blood loss, pathology, NSCLC staging, and ASA score.

Table 2 Correlation of non-restricted PRISm with PPC compared to mild COPD

| Groups | PPC | |
| --- | --- | --- |
|  | OR (95%CI) | *P* value |
| Mild COPD | Reference |  |
| Non-restricted PRISm | 1.32 (0.45, 3.88) | 0.61 |

Abbreviations: PRISm, preserved ratio impaired spirometry; PPC, postoperative pulmonary complication; mild COPD, chronic obstructive pulmonary disease, forced expiratory volume in one second≥80% predicted; OR, odd ratio; CI, confidence interval.

Adjusted for sex, age, BMI, history of smoking, pack-years, hypertension, diabetes, largest tumor dimension, tumor location, surgical approach, type of surgery, operative duration, blood loss, pathology, NSCLC staging, and ASA score.

Table 3 Correlation of non-restricted PRISm with PPC compared to moderate COPD

| Groups | PPC | |
| --- | --- | --- |
|  | OR (95%CI) | *P* value |
| Moderate COPD | Reference |  |
| Non-restricted PRISm | 0.88 (0.38, 2.03) | 0.77 |

Abbreviations: PRISm, preserved ratio impaired spirometry; PPC, postoperative pulmonary complication; moderate COPD, chronic obstructive pulmonary disease, 50% predicted≦forced expiratory volume in one second<80% predicted; OR, odd ratio; CI, confidence interval.

Adjusted for sex, age, BMI, history of smoking, pack-years, hypertension, diabetes, largest tumor dimension, tumor location, surgical approach, type of surgery, operative duration, blood loss, pathology, NSCLC staging, and ASA score.

Table 4 Correlation of non-restricted PRISm with PPC compared to severe COPD

| Groups | PPC | |
| --- | --- | --- |
|  | OR (95%CI) | *P* value |
| Severe COPD | Reference |  |
| Non-restricted PRISm | 0.065 (0.003, 1.377) | 0.079 |

Abbreviations: PRISm, preserved ratio impaired spirometry; PPC, postoperative pulmonary complication; severe COPD, chronic obstructive pulmonary disease, forced expiratory volume in one second<50% predicted; OR, odd ratio; CI, confidence interval.

Adjusted for sex, age, BMI, history of smoking, pack-years, hypertension, diabetes, largest tumor dimension, tumor location, surgical approach, type of surgery, operative duration, blood loss, pathology, NSCLC staging, and ASA score.

Table 5 Correlation of restricted PRISm with PPC compared to COPD

| Groups | PPC | |
| --- | --- | --- |
|  | OR (95%CI) | *P* value |
| COPD | Reference |  |
| Restricted PRISm | 1.104 (0.501,2.433) | 0.806 |

Abbreviations: PRISm, preserved ratio impaired spirometry; PPC, postoperative pulmonary complication; COPD, chronic obstructive pulmonary disease; OR, odd ratio; CI, confidence interval.

Adjusted for sex, age, BMI, history of smoking, pack-years, hypertension, diabetes, largest tumor dimension, tumor location, surgical approach, type of surgery, operative duration, blood loss, pathology, NSCLC staging, and ASA score.

Table 6 Correlation of restricted PRISm with PPC compared to mild COPD

| Groups | PPC | |
| --- | --- | --- |
|  | OR (95%CI) | *P* value |
| Mild COPD | Reference |  |
| Restricted PRISm | 0.928 (0.422, 2.037) | 0.851 |

Abbreviations: PRISm, preserved ratio impaired spirometry; PPC, postoperative pulmonary complication; mild COPD, chronic obstructive pulmonary disease, forced expiratory volume in one second≥80% predicted; OR, odd ratio; CI, confidence interval.

Adjusted for sex, age, BMI, history of smoking, pack-years, hypertension, diabetes, largest tumor dimension, tumor location, surgical approach, type of surgery, operative duration, blood loss, pathology, NSCLC staging, and ASA score.

Table 7 Correlation of restricted PRISm with PPC compared to moderate COPD

| Groups | PPC | |
| --- | --- | --- |
|  | OR (95%CI) | *P* value |
| Moderate COPD | Reference |  |
| Restricted PRISm | 0.460 (0.224, 0.942) | **0.034** |

Abbreviations: PRISm, preserved ratio impaired spirometry; PPC, postoperative pulmonary complication; moderate COPD, chronic obstructive pulmonary disease, 50% predicted≦forced expiratory volume in one second<80% predicted; OR, odd ratio; CI, confidence interval.

Adjusted for sex, age, BMI, history of smoking, pack-years, hypertension, diabetes, largest tumor dimension, tumor location, surgical approach, type of surgery, operative duration, blood loss, pathology, NSCLC staging, and ASA score.

Table 8 Correlation of restricted PRISm with PPC compared to severe COPD

| Groups | PPC | |
| --- | --- | --- |
|  | OR (95%CI) | *P* value |
| Severe COPD | Reference |  |
| Restricted PRISm | 0.031 （0.003, 0.366) | **0.006** |

Abbreviations: PRISm, preserved ratio impaired spirometry; PPC, postoperative pulmonary complication; severe COPD, chronic obstructive pulmonary disease, forced expiratory volume in one second<50% predicted; OR, odd ratio; CI, confidence interval.

Adjusted for sex, age, BMI, history of smoking, pack-years, hypertension, diabetes, largest tumor dimension, tumor location, surgical approach, type of surgery, operative duration, blood loss, pathology, NSCLC staging, and ASA score.
