# Supplementary material for: Association between preserved ratio impaired spirometry and postoperative pulmonary complications in patients undergoing lung cancer resection
Source: Front Med (Lausanne). 2026 Oct 14;13:1803143. doi: 10.3389/fmed.2026.1803143 (PMC13713789; doi:10.3389/fmed.2026.1803143)
Supplement: Supplementary file 2 [file Table2.docx]

Supplementary Table S1. Univariable logistic regression and event distribution of variables included in the final multivariable model (PRISm group vs. normal spirometry group)

|  | Number of PPCs | Crude OR | 95% CI | *P* value |
| --- | --- | --- | --- | --- |
| Age (years) | - | 1.011 | 0.987-1.035 | 0.366 |
| BMI (kg/m^2^) | - | 1.002 | 0.931-1.078 | 0.952 |
| Male | 42 (36.2%) | 0.651 | 0.403-1.052 | 0.080 |
| Pack-year | - | 1.009 | 0.990-1.029 | 0.343 |
| History of smoking | 11 (34.4%) | 1.241 | 0.575 -2.678 | 0.582 |
| Hypertension | 19 (21.8%) | 0.569 | 0.321-1.008 | 0.053 |
| Diabetes | 11 (33.3%) | 1.179 | 0.549-2.531 | 0.672 |
| Surgical approach |  | NA* |  |  |
| VATS/RATS | 102 (29.9%) |  |  |  |
| Open thoracotomy | 1 (100.0%) |  |  |  |
| Type of surgery |  |  |  | **0.003** |
| Lobectomy | 74 (37.4%) | 1,00 (reference) |  |  |
| Bilobectomy | 27 (19.7%) | 1.492 | 0.282-7.885 | 0.638 |
| Others | 2 (28.6%) | 0.614 | 0.113 -3.336 | 0.572 |
| Operative duration (hours) | - | 1.467 | 1.038-2.073 | **0.030** |
| Blood loss (mL) | - | 1.000 | 0.997-1.003 | 0.934 |
| Largest tumor dimension (mm) | - | 1.061 | 1.037-1.086 | **<0.001** |
| Tumor location |  |  |  |  |
| Right | 67 (28.9%) | 1.00 (reference) |  |  |
| Left | 36 (32.7%) | 1.198 | 0.735-1.954 | 0.469 |
| Pathology |  |  |  | **0.015** |
| Adenocarcinoma | 84 (27.6%) | 1.00 (reference) |  |  |
| Squamous Cell | 11 (44.0%) | 0.239 | 0.076-0.750 | 0.014 |
| others | 8 (61.5%) | 0.491 | 0.125-1.929 | 0.308 |
| NSCLC staging |  |  |  | **<0.001** |
| Tis/I | 19 (57.6%) | 1.00 (reference) |  |  |
| II | 78 (29.2%) | 0.142 | 0.027-0.745 | 0.021 |
| III/ IV | 6 (14.3%) | 0.543 | 0.092-3.217 | 0.501 |
| ASA score≦2 | 103 (30.1%) | NA* |  |  |

*NA = not estimable.

Abbreviations: PRISm, preserved ratio impaired spirometry; BMI, body mass index; 1 pack-year is defined as 20 cigarettes smoked every day for 1 year; VATS, video-assisted thoracic surgery; RATS, robotic-assisted thoracic surgery; NSCLC, non-small cell lung cancer; ASA, American Society of Anesthetist; PPC, postoperative pulmonary complications; OR, odds ratio; CI, confidence interval.
