# Supplementary material for: Association between preserved ratio impaired spirometry and postoperative pulmonary complications in patients undergoing lung cancer resection
Source: Front Med (Lausanne). 2026 Oct 14;13:1803143. doi: 10.3389/fmed.2026.1803143 (PMC13713789; doi:10.3389/fmed.2026.1803143)
Supplement: Supplementary file 3 [file Table3.docx]

### **Supplementary Table S2. Univariable logistic regression of candidate covariates for PPC**

| **Variable** | **PRISm vs Normal OR (95%CI)** | ***P*** | **PRISm vs COPD OR (95%CI)** | ***P*** |
| --- | --- | --- | --- | --- |
| Age (per year) | 1.01 (0.99-1.04) | 0.362 | 1.02 (1.00-1.04) | 0.077 |
| Male | 1.54 (0.95-2.48) | 0.081 | 1.94 (1.29-2.92) | 0.001 |
| BMI (per kg/m^2^) | 1.00 (0.93-1.08) | 0.952 | 0.92 (0.86-0.99) | 0.016 |
| Ever smoking | 1.24 (0.58-2.68) | 0.585 | 1.19 (0.72-1.97) | 0.506 |
| Pack-years | 1.01 (0.99-1.03) | 0.350 | 1.00 (0.99-1.01) | 0.478 |
| Hypertension | 0.57 (0.32-1.01) | 0.047 | 0.93 (0.60-1.46) | 0.760 |
| Diabetes | 1.18 (0.55-2.53) | 0.674 | 0.89 (0.43-1.82) | 0.739 |
| Open thoracotomy | NA (separation) | 0.121 | 8.80 (1.07-72.22) | 0.011 |
| Sub-lobar resection (vs lobectomy) | 0.41 (0.25-0.68) | 0.001 | 0.36 (0.23-0.56) | <0.001 |
| Sleeve lobectomy (vs lobectomy) | NA (separation) | 0.001 | 1.76 (0.51-5.99) | <0.001 |
| Pneumonectomy (vs lobectomy) | 3.35 (0.30-37.60) | 0.001 | 4.39 (0.51-38.16) | <0.001 |
| Operative duration (per hour) | 1.47 (1.04-2.07) | 0.030 | 1.72 (1.27-2.34) | <0.001 |
| Blood loss (per mL) | 1.00 (1.00-1.00) | 0.934 | 1.00 (1.00-1.01) | 0.021 |
| Largest tumor dimension (per mm) | 1.06 (1.04-1.09) | <0.001 | 1.05 (1.03-1.07) | <0.001 |
| Site: RML (vs RUL) | 0.46 (0.17-1.28) | 0.011 | 0.61 (0.26-1.44) | 0.093 |
| Site: RLL (vs RUL) | 2.27 (1.20-4.28) | 0.011 | 1.82 (1.01-3.25) | 0.093 |
| Site: LUL (vs RUL) | 1.19 (0.62-2.28) | 0.011 | 0.97 (0.57-1.65) | 0.093 |
| Site: LLL (vs RUL) | 1.76 (0.85-3.66) | 0.011 | 1.36 (0.75-2.49) | 0.093 |
| Squamous cell (vs adenocarcinoma) | 2.06 (0.90-4.71) | 0.014 | 3.06 (1.73-5.42) | <0.001 |
| Other pathology (vs adenocarcinoma) | 5.24 (1.54-17.85) | 0.014 | 1.50 (0.69-3.25) | <0.001 |
| Stage II (vs Tis/I) | 3.83 (1.83-8.00) | <0.001 | 2.58 (1.30-5.11) | 0.002 |
| Stage III/IV (vs Tis/I) | 7.06 (1.34-37.11) | <0.001 | 5.56 (1.16-26.55) | 0.002 |

*P* values for categorical variables are overall likelihood-ratio test *P* values. NA (separation): level not estimable due to zero cells. Variable selection: covariates with *P* < 0.20 were retained in the corresponding multivariable model in addition to the forced set.
